# Hippocampal ΔFosB expression is associated with cognitive impairment in a subgroup of patients with childhood epilepsies

**DOI:** 10.3389/fneur.2023.1331194

**Published:** 2024-01-11

**Authors:** Chia-Hsuan Fu, Jason C. You, Carrie Mohila, Robert A. Rissman, Daniel Yoshor, Angela N. Viaene, Jeannie Chin

**Affiliations:** ^1^Department of Neuroscience, Baylor College of Medicine, Houston, TX, United States; ^2^Department of Pathology, Texas Children's Hospital and Baylor College of Medicine, Houston, TX, United States; ^3^Department of Neurosciences, University of California San Diego School of Medicine, La Jolla, CA, United States; ^4^Veteran's Affairs (VA) San Diego Healthcare System, San Diego, CA, United States; ^5^Department of Neurosurgery, Baylor College of Medicine, Houston, TX, United States; ^6^Department of Pathology and Laboratory Medicine, The Children's Hospital of Philadelphia, Philadelphia, PA, United States; ^7^Department of Pathology and Laboratory Medicine, Perelman School of Medicine, University of Pennsylvania, Philadelphia, PA, United States

**Keywords:** dentate gyrus, Alzheimer's disease, seizures, epigenetic, epilepsy, intellectual disability, deltaFosB, cognition

## Abstract

Epilepsy is a chronic neurological disorder characterized by recurrent seizures, and is often comorbid with other neurological and neurodegenerative diseases, such as Alzheimer's disease (AD). Patients with recurrent seizures often present with cognitive impairment. However, it is unclear how seizures, even when infrequent, produce long-lasting deficits in cognition. One mechanism may be seizure-induced expression of ΔFosB, a long-lived transcription factor that persistently regulates expression of plasticity-related genes and drives cognitive dysfunction. We previously found that, compared with cognitively-intact subjects, the activity-dependent expression of ΔFosB in the hippocampal dentate gyrus (DG) was increased in individuals with mild cognitive impairment (MCI) and in individuals with AD. In MCI patients, higher ΔFosB expression corresponded to lower Mini-Mental State Examination scores. Surgically resected DG tissue from patients with temporal lobe epilepsy also showed robust ΔFosB expression; however, it is unclear whether ΔFosB expression also corresponds to cognitive dysfunction in non-AD-related epilepsy. To test whether DG ΔFosB expression is indicative of cognitive impairment in epilepsies with different etiologies, we assessed ΔFosB expression in surgically-resected hippocampal tissue from 33 patients with childhood epilepsies who had undergone Wechsler Intelligence Scale for Children (WISC) testing prior to surgery. We found that ΔFosB expression is inversely correlated with Full-Scale Intelligence Quotient (FSIQ) in patients with mild to severe intellectual disability (FSIQ < 85). Our data indicate that ΔFosB expression corresponds to cognitive impairment in epilepsies with different etiologies, supporting the hypothesis that ΔFosB may epigenetically regulate gene expression and impair cognition across a wide range of epilepsy syndromes.

## 1 Introduction

Epilepsy is one of the most common neurological diseases and affects people of all ages (1, 2). There is often disrupted consciousness and memory during a seizure, but recurrent seizures can also lead to long-lasting changes in neuronal and network function, and drive chronic impairments in cognition that persist even during seizure-free periods (3–5). Notably, cognitive impairment can develop even with infrequent seizures (6, 7). Seizures are frequently co-morbid with other neurological and neurodegenerative diseases, such as Alzheimer's disease (AD), Down syndrome, autism, Fragile X syndrome, and others, and seizure-induced cognitive dysfunction may also contribute to or exacerbate cognitive deficits observed in those neurological disorders (8–17). Thus, in addition to improving methods of seizure control, it is also critical to understand the molecular and network mechanisms that underlie cognitive impairment in epilepsy, and in particular, long-lasting mechanisms that may be engaged even when seizures are infrequent.

One molecular mechanism that may contribute to such long-lasting effects on cognition is the activity-induced expression of ΔFosB, a highly stable transcription factor in the immediate early gene family, in the hippocampal dentate gyrus (DG). ΔFosB has an unusually long half-life of roughly 8 days *in vivo*, allowing it to accumulate within the nucleus even with relatively infrequent repetitive activation of neurons (18). ΔFosB expression is robustly induced in the nucleus accumbens after exposure to drugs of abuse, and accumulates in the hippocampus following recurrent seizures (19–21). Notably, ΔFosB recruits histone modifying enzymes to epigenetically regulate target gene expression, resulting in long-lasting control of gene expression even after the initial activating stimulus is over (18, 22). In various brain regions, ΔFosB binds to a multitude of gene targets, including those related to neuronal excitability and plasticity (20, 23, 24). Neuronal activity-dependent accumulation of ΔFosB within hippocampal neurons following repeated seizure activity thus chronically alters gene expression and can affect cognitive processes. Indeed, we have previously shown that ΔFosB is robustly induced in dentate granule neurons after seizure activity in mouse models for studying epilepsy or for studying AD, which is accompanied by a high incidence of epilepsy (19, 25). In those studies, ΔFosB expression directly corresponded to cognitive impairment, and inhibition of ΔFosB activity improved cognition (19, 25).

The relevance of ΔFosB to human disease is supported by findings that its expression is increased robustly in the DG of individuals with temporal lobe epilepsy (TLE), AD, or mild cognitive impairment (MCI; often considered prodromal AD) (25). Moreover, in patients with MCI, increasing magnitudes of ΔFosB expression corresponded to poorer performance on the Mini-Mental State Examination (MMSE) test of cognition (25), suggesting that ΔFosB may function similarly in humans as in mouse models of disease.

However, it is unclear whether neuronal activity-dependent ΔFosB expression in the DG also reflects cognitive impairment in patients with epilepsy outside the context of AD, or in patients with epilepsy who develop seizures at younger ages. To assess this possibility, we obtained resected hippocampal DG samples from patients with childhood epilepsies who had undergone neuropsychiatric assessment prior to hippocampectomy, and assessed whether ΔFosB expression in human DG is related to any measures of cognitive function in these patients. We found that DG ΔFosB expression corresponds to decreased Full-Scale Intelligence Quotient (FSIQ), a measure of cognitive ability in children, in patients with borderline to poor intellectual functioning.

## 2 Materials and methods

### 2.1 Human tissue

Fixed DG samples from 33 individuals with childhood epilepsies were obtained from hippocampectomy specimens obtained after surgical resection for treatment of epilepsy at the Children's Hospital of Philadelphia (Philadelphia, PA) between 2000 and 2019. Seven of the 33 samples were obtained from patients who underwent selective hippocampectomies. The remaining 26 samples were obtained from patients who underwent either surgical excision of extra-hippocampal lesions in addition to the hippocampectomy, or temporal lobectomy with the hippocampus being removed as a unique surgical specimen. All samples were formalin-fixed, processed, paraffin-embedded, and sectioned at 5 μm. Clinical information was retrospectively collected from the electronic medical record in accordance with the Children's Hospital of Philadelphia Institutional Review Board (protocol IRB 19-016521).

Fixed DG samples from adult control individuals or individuals with MCI, AD, or TLE were from previously published patient cohorts (25). Briefly, fixed post-mortem DG samples from individuals with AD or MCI and age-matched controls were obtained from the Alzheimer's Disease Research Center at the University of California San Diego (San Diego, CA), and sectioned at 60 μm. Fixed surgically-resected DG samples from individuals with TLE were obtained and used with informed consent under Institutional Review Board protocol H-10255; samples were resection specimens derived from surgery for epilepsy in adult patients treated at Baylor College of Medicine (Houston, TX).

### 2.2 Immunohistochemistry

Fixed DG samples derived from surgical resections of the hippocampus in patients with childhood epilepsies were deparaffinized and rehydrated following a standard procedure: three 5-min rinses in xylenes, two 10-min rinses in 100% ethanol, two 10-min rinses in 95% ethanol, and then two 5-min rinses in distilled water. Sections then underwent alternating rinses with PBS and PBS with 0.5% Triton-X (PBS-Tx-0.5%) in between the following steps: (1) 15-min incubation with endogenous peroxidase blocking solution consisting of 3% hydrogen peroxide, 10% methanol, and PBS; (2) 10-min antigen retrieval with citrate buffer at 85°C; (3) 10-min incubation in 90% formic acid; (4) 60-min incubation with a non-specific blocking solution consisting of 10% normal goat serum (Vector Laboratories, Cat# S-1000, RRID:AB_2336615), 1% blocking grade non-fat dry milk (Bio-Rad, Cat# 1706404), 0.2% gelatin (Sigma-Aldrich, Cat# G2500), and PBS-Tx 0.5%; (5) overnight primary antibody incubation at 4°C; (6) 60-min secondary antibody incubation; (7) 60-min incubation with avidin-biotin complex (Vectastain, Cat# PK-6100), and (8) 10-min development with diaminobenzidine (Vector Laboratories, Cat# SK-4103, RRID:AB_2336521). The antibody concentrations used were 1:200 for rabbit anti-ΔFosB antibody (Cell Signaling, Cat# 14695, RRID:AB_2798577) and 1:200 for goat anti-rabbit biotinylated antibody (Vector Laboratories, Cat# BA-1000, RRID:AB_2313606).

### 2.3 Imaging and analysis

Immunostained sections were imaged by the RNA *in situ* Hybridization Core facility at Baylor College of Medicine. Analysis was performed using Fiji ImageJ (NIH, RRID:SCR_002285). For quantification of DG ΔFosB expression, images were first converted to 16-bit black and white images. For each patient sample, quantification was performed on 20 randomly selected dentate granule cells following previously published procedures, which we had found allowed for reliable representation of ΔFosB expression in the human DG (25). The mean pixel intensity for each dentate granule cell was measured. The average of the mean pixel intensities of three nearby acellular white matter tract areas was used for background correction. Immunoreactivity (IR) was defined as the average of the mean pixel intensities for the 20 dentate granule cells, corrected for background. Quantification was performed by an experimenter blind to the specific diagnoses and neuropsychiatric testing scores of each patient.

### 2.4 Statistics

Statistical analyses were performed using Prism 10 (GraphPad, RRID:SCR_002798). Differences between two groups were assessed via two-tailed unpaired Student's *t*-tests. Correlations were assessed via simple regression analyses. *P*-value correction for multiple comparisons were performed with the Holm-Sidak *post-hoc* test.

## 3 Results

### 3.1 Patient demographics

We obtained surgically resected hippocampal tissue from 33 patients with childhood epilepsies who had been administered the Wechsler Intelligence Scale for Children, Fourth Edition (WISC-IV) assessment prior to hippocampectomy (Table 1). There were similar numbers of male (48.5%) and female (51.5%) patients, and patient ages ranged from 4.58 to 20.58 years old. All 33 patients were tested prior to hippocampal resection, with the interval between neuropsychiatric assessment and surgery varying from 1 month to almost 5 years.

**Table 1:** Patient demographic information.

	**Childhood epilepsy cohort**
**Sex**	**# patients (% patients)**
Male	16 (48.5%)
Female	17 (51.5%)
**Age (years)**	**Mean** **±SD (range)**
At hippocampectomy	12.74 ± 4.05 (4.58–20.58)
At neuropsychiatric testing	11.71 ± 4.05 (4.50–19.92)
Difference	1.03 ± 1.16 (0.08–4.83)
**Seizure onset (22/33 patients)**	**Mean** **±SD (range)**
Age (years)	5.08 ± 3.66 (0.00–13.00)
Years with seizures prior to hippocampectomy	7.60 ± 4.06 (1.08–15.92)
**Seizure frequency (19/33 patients)**	**Mean** **±SD (range)**
Seizures per month	68.7 ± 131.2 (0.25–532)
**Neuropathological diagnoses**	**# patients (% patients)**
Encephalitis	5 (15.2%)
Tumor	5 (15.2%)
Infarction	4 (12.1%)
Focal cortical dysplasia	3 (9.1%)
Sturge-Weber syndrome	1 (3%)
**Neuropsychiatric diagnoses^#^**	**# patients (% patients)**
Attention-deficit/hyperactivity disorder	8 (24.2%)
Asperger's syndrome	1 (3%)
**WISC-IV score**	**Mean** **±SD (range)**
Full-Scale Intelligence Quotient	80.4 ± 17.0 (46–105)^*^
General Ability (7/33 patients)	88.4 ± 15.7 (64–113)
Verbal Comprehension (28/33 patients)	86.7 ± 14.1 (50–116)
Perceptual Reasoning (24/33 patients)	87.4 ± 16.4 (51–112)
Working Memory (23/33 patients)	82.7 ± 16.5 (55–113)
Processing Speed (26/33 patients)	81.3 ± 18.2 (45–119)

Demographic information regarding patient sex, age, seizure onset, seizure frequency, co-occurrence of other neuropathological and psychiatric diagnoses, and scores on the Wechsler Intelligence Scale for Children-Fourth Edition (WISC-IV) cognitive assessment. For categories in which information is not available for all 33 patients, the number of patients for which the information is available is indicated in parentheses.

# Neuropsychiatric diagnoses reflect what was documented in patients' medical records; some terminology may be outdated.

* Full-Scale Intelligence Quotient (FSIQ) of patients in the childhood epilepsy cohort is significantly decreased (p = 0.0306, two-tailed unpaired Student's t-test) compared with the general population (mean = 100, SD = 15).

SD, standard deviation.

Of the 33 patients, 23 patients exhibited only focal seizures, six patients exhibited focal seizures with secondary generalization, one patient exhibited only generalized tonic-clonic seizures, and three patients exhibited both focal and generalized seizures. Of the 32 patients who experienced focal seizures, 24 patients had seizures with impaired awareness (complex partial seizures), one patient exhibited focal seizures without impaired awareness (simple partial seizures), and seven patients were unspecified. Four patients had focal seizures secondary to lesions.

Information about seizure history, including age at seizure onset and seizure frequency, was available only for a portion of the patients (19–22 of the 33 patients included in this study). Of the patients with these data available, age at seizure onset was 5.08 ± 3.66 (mean ± SD) years, with variation ranging from within the 1st year of life to 13 years of age. Patients exhibited seizures for 7.6 ± 4.06 (mean ± SD) years prior to resection. The frequency of the seizures that patients presented with ranged from three seizures per year to 15–20 seizures per day.

While etiology of epilepsy was unclear for the majority of cases in this study, there were patients who received clinical diagnoses that have known associations with seizures, including encephalitis (26, 27), tumor (28, 29), infarction (30, 31), focal cortical dysplasia (32, 33), and Sturge-Weber syndrome (34, 35). In addition, 24.2% (8/33) of the patients had psychiatric diagnoses of attention-deficit/hyperactivity disorder (ADHD), with one patient also having Asperger's syndrome, which are comorbidities that have bidirectional relationships with epilepsy (36–39). 30.3% of patients (10/33) did not have additional neuropathological or psychiatric diagnoses.

All patients underwent neuropsychiatric testing prior to hippocampectomy in the form of the WISC-IV. WISC testing is composed of subtests that fall under four broad indices of intellectual functioning, including verbal comprehension, perceptual reasoning, working memory, and processing speed (40). Scores from verbal comprehension and perceptual reasoning subtests constitute the general ability index, while scores from all four indices constitute the Full-Scale Intelligence Quotient (FSIQ) (41, 42). FSIQ is considered a global assessment of cognitive functioning. While documented FSIQ scores were available for all patients in this study, the scores for the individual indices were not available for all patients. The average FSIQ for the general population is 100, with a standard deviation (SD) of 15, and usually ranges from 40 (exceptionally low) to 160 (exceptionally superior) (40). Notably, the average FSIQ of patients with childhood epilepsies included in this study was 80.4 with a SD of 17.0, which is significantly lower than that of the general population (80.4 ± 17 vs. 100 ± 15; *p* = 0.031, two-tailed unpaired Student's *t*-test). Patients who also received an ADHD diagnosis had lower average FSIQ compared with patients who did not receive an ADHD diagnosis (68.38 ± 14.50 vs. 84.28 ± 16.16; *p* = 0.0189, two-tailed unpaired Student's *t*-test), which is consistent with prior findings in the literature (43, 44).

### 3.2 ΔFosB expression in the DG in childhood epilepsy patients is similar to that in patients with TLE, MCI, or AD

**Figure 1:**
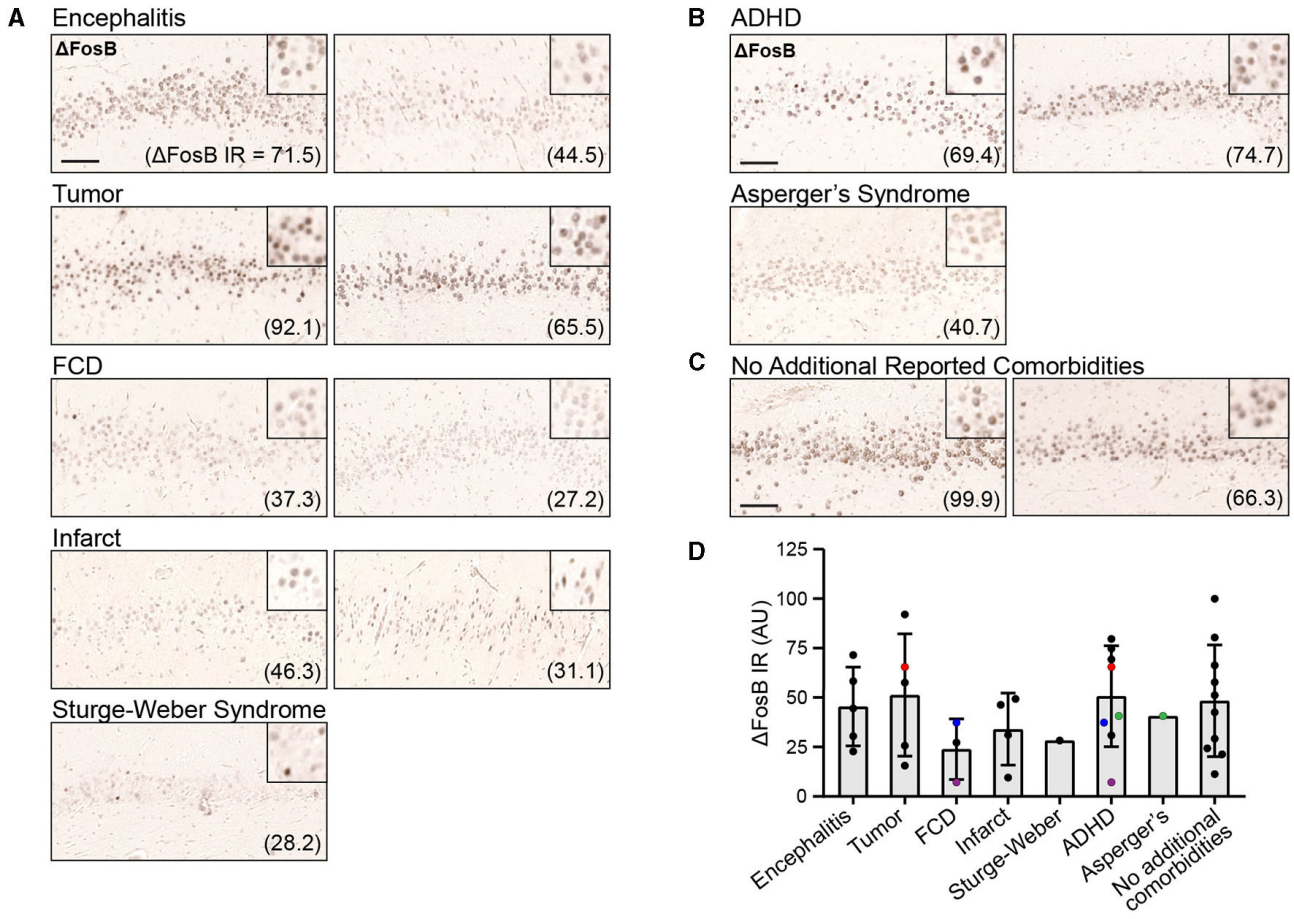
Dentate gyrus (DG) ΔFosB immunoreactivity (IR) in patients with childhood epilepsies. **(A, B)** Example images of DG ΔFosB IR in surgically resected hippocampal tissue from patients with childhood epilepsies who presented with additional neuropathological **(A)** and neuropsychiatric diagnoses **(B)**, and from patients without reported comorbidities **(C)**. Quantification of DG ΔFosB IR in arbitrary units (AU) is indicated in parentheses for each patient. **(D)** DG ΔFosB IR quantification for all 33 patients grouped by neuropathological and neuropsychiatric diagnoses. Colored (red, blue, purple, and green) data points indicate patients who had received multiple diagnoses and were therefore represented multiple times in the graph. Scale bar: 100 μm.

To assess whether ΔFosB is expressed in childhood epilepsy syndromes as it is in adult TLE, MCI, and AD, and whether its expression is related to cognitive function in epilepsy, we first performed immunohistochemistry for ΔFosB on DG samples from these 33 patients (Figure 1; Supplementary Figure 1). We observed distinct nuclear expression of ΔFosB in dentate granule cells, consistent with the pattern observed in animal models with epilepsy and previous studies of human samples (25). We noted that the intensity of ΔFosB expression varied between patients, and this variability was reflected in the quantification of ΔFosB immunoreactivity (indicated by arbitrary units in parentheses; Figure 1D). However, there was no systematic difference in ΔFosB expression between patients with or without additional neuropathological or psychiatric diagnoses in the present dataset (Supplementary Figure 2). In addition, although DG ΔFosB expression in mice corresponds to seizure frequency, DG ΔFosB expression in this cohort of patients with childhood epilepsies did not directly correspond to either seizure frequency (*N* = 19, *R*^2^ = 0.087, *p* = 0.219) or number of years patients experienced seizures prior to hippocampectomy (*N* = 22, *R*^2^ = 0.002, *p* = 0.839). However, these data were not available for all 33 patients.

**Figure 2:**
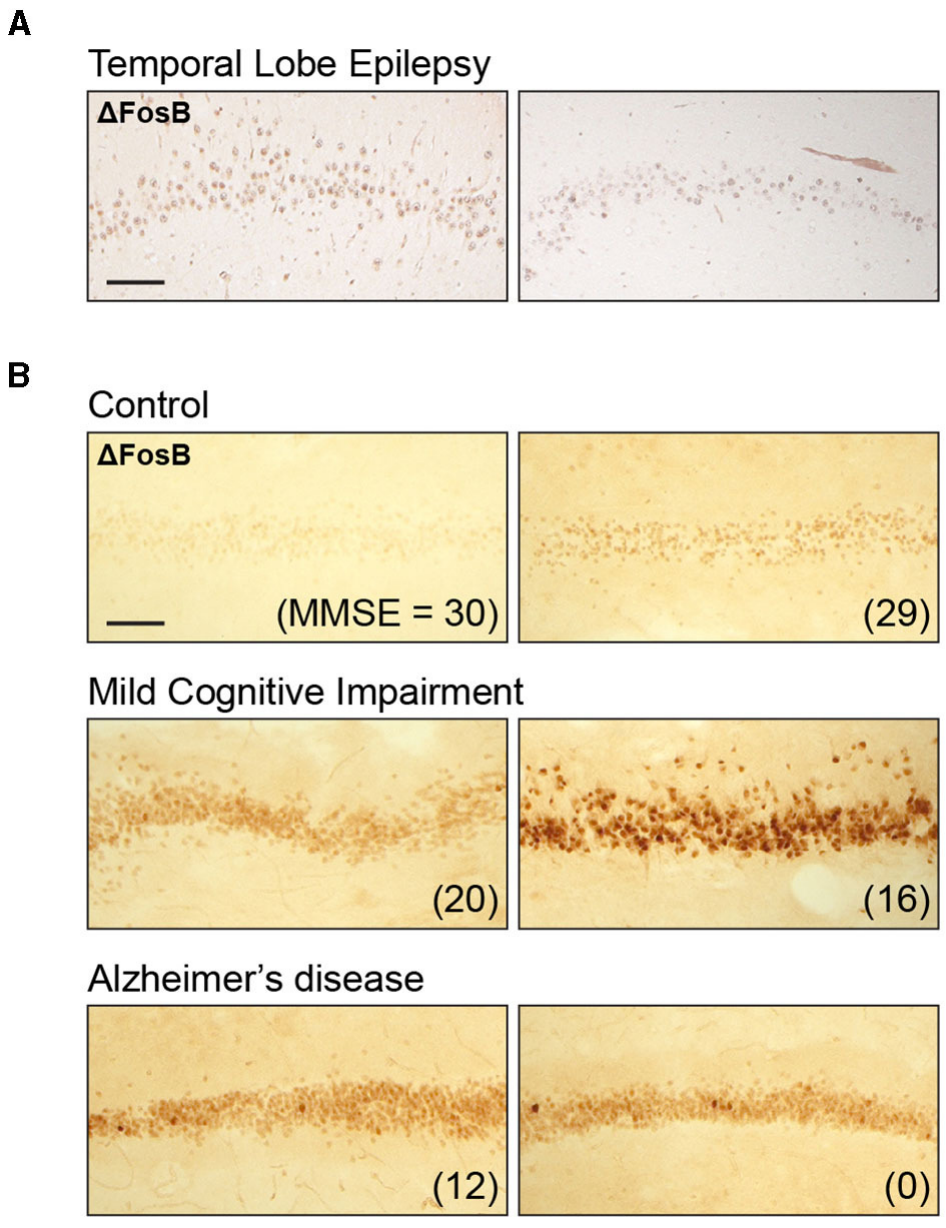
Dentate gyrus ΔFosB immunoreactivity in adult individuals with temporal lobe epilepsy (TLE), mild cognitive impairment (MCI), or Alzheimer's disease (AD). **(A)** Example images of dentate gyrus ΔFosB immunoreactivity in surgically resected tissue from two patients with TLE. **(B)** Example images of dentate gyrus ΔFosB immunoreactivity in postmortem samples from control individuals, individuals with MCI, and individuals with AD. Mini-Mental State Examination (MMSE) scores are indicated in parentheses for each patient. Sections from patients included in this figure were stained as part of a previously published study (25); examples shown here are original, previously unpublished images. Scale bar: 100 μm.

To assess whether the DG ΔFosB expression pattern in patients with childhood epilepsies is qualitatively similar to the expression pattern in patients with TLE, we revisited ΔFosB expression patterns in hippocampal resection tissues obtained from adult patients with TLE in a previous study (25). Similar to our findings in patients with childhood epilepsies, ΔFosB expression in adult patients with TLE showed a nuclear pattern, with clearly defined small circular areas of intense staining, particularly in comparison with the diffuse background staining observed in the surrounding brain parenchyma (Figure 2A). This result indicates that DG ΔFosB expression is clearly observed in both childhood and adult epilepsies.

In our previous study demonstrating robust ΔFosB expression in adult TLE, we did not have neuropsychiatric data to assess the relationship between ΔFosB and cognitive function in those individuals. However, we were able to assess the relationship between ΔFosB expression and cognition in individuals with MCI or AD, which is associated with an increase in seizure incidence (45–48). Recent studies demonstrated that seizure activity tends to begin early in disease progression and is associated with earlier and faster rate of cognitive decline (13, 16, 47, 49). In our previous study, we found that ΔFosB expression in the DG was increased in individuals with either MCI or AD compared with control individuals, as shown in Figure 2B. We noted that the staining pattern in the MCI and AD groups was also nuclear, similar to the epilepsy samples (Figure 2B). Of particular relevance to this study, DG ΔFosB expression did not correspond to Mini-Mental State Examination (MMSE) scores in control individuals or in AD patients with severe cognitive impairments, but ΔFosB expression did correspond to MMSE scores in MCI patients, indicating a relationship between DG ΔFosB expression and cognitive dysfunction in earlier or milder stages of AD (25).

### 3.3 ΔFosB expression in the DG of patients with childhood epilepsies corresponds to FSIQ in patients with borderline to poor intellectual functioning

To determine whether DG ΔFosB expression is related to cognitive function in patients with childhood epilepsies, we compared ΔFosB expression levels with FSIQ, a global measure of cognitive functioning. Because we found no relationship between ΔFosB and MMSE scores in control individuals but found a negative relationship in MCI patients in which higher ΔFosB expression reflected poorer cognitive function (25), we divided the childhood epilepsy cohort based on cognitive function, as defined by FSIQ. We used a FSIQ cutoff of 85, above which children are typically considered to have average or above average intellectual functioning, and below which children are considered to have borderline intellectual functioning (FSIQ > 70) or intellectual disability (FSIQ < 70) (50).

**Figure 3:**
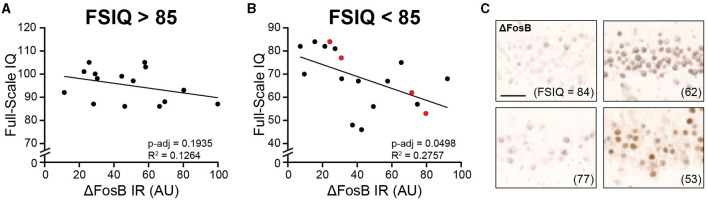
Dentate gyrus ΔFosB immunoreactivity corresponds to Full-Scale Intelligence Quotient (FSIQ) in patients with childhood epilepsies and borderline intellectual functioning or intellectual disability. **(A, B)** Regression analyses of dentate gyrus ΔFosB immunoreactivity and FSIQ for patients with FSIQ > 85 **(A)** and patients with FSIQ < 85 **(B)**. **(C)** Representative images of dentate gyrus ΔFosB immunoreactivity of patients indicated in red in **(B)**. FSIQ is indicated in parentheses for each patient. IR, immunoreactivity; AU, arbitrary units. Scale bar: 50 μm.

We found that in individuals with FSIQ > 85, ΔFosB did not correspond to FSIQ (Figure 3A). However, in individuals with FSIQ < 85, higher levels of ΔFosB expression corresponded to lower FSIQ (Figures 3B, C). There was no significant relationship between any individual index score with ΔFosB in either group, which may in part be due to variable sample sizes since not all index scores were available for every patient (Supplementary Figure 3). While not statistically significant, we noted that in individuals with FSIQ < 85, the general trend for all indices were negative (i.e., decreased scores with increased ΔFosB; Supplementary Figure 3B), whereas the general trends for individuals with FSIQ > 85 were more mixed (Supplementary Figure 3A). Subdividing patients by sex, time between neuropsychiatric testing and hippocampectomy, and other neuropathological and psychiatric diagnoses did not yield other significant relationships (Supplementary Figure 4). Interestingly, while scores for most indices showed no or negative trends with ΔFosB, the processing speed index score showed positive trends with ΔFosB in several subdivisions of patients (Supplementary Figures 3–5), and the trend was significant in patients whose tissue was found to have hippocampal sclerosis (Supplementary Figure 5F).

## 4 Discussion

In summary, we found that ΔFosB is expressed robustly in the dentate granule cells of patients with childhood epilepsies, similar to adult individuals with TLE, MCI, or AD, and that the magnitude of ΔFosB expression in these cells corresponded to FSIQ in patients whose FSIQ is < 85.

Our finding that ΔFosB is expressed similarly in the DG of humans as in that of mice support the potential translatability of the functions and mechanisms of action of ΔFosB that have been uncovered in rodent models of human diseases. An FSIQ of 85 has been used as the cutoff between individuals with average intellectual functioning and those with borderline intellectual functioning (BIF; FSIQ 70–84) or intellectual disability (FSIQ < 70) (50). While BIF is not considered a mental disability in the most recent Diagnostic and Statistical Manual of Mental Disorders (DSM-5), children with BIF have high risk for the same mental, social, and intellectual difficulties as those with intellectual disability (50–54). Our finding that ΔFosB corresponds to FSIQ in this patient subpopulation (FSIQ < 85) suggests that in these individuals, ΔFosB may be engaging mechanisms that negatively affect cognition. It has been shown in rodent models that alterations to ΔFosB expression in the hippocampus in non-disease conditions are sufficient to induce hippocampal-dependent learning and memory deficits, whereas normalizing aberrantly increased ΔFosB activity in disease conditions improves cognition (19, 21, 25, 55). Additionally, due the long half-life ΔFosB, its impact on cognition could persist even during periods in between seizures. Thus, the findings in this study suggest that in patients with FSIQ < 85, achieving seizure control may not be sufficient, and that it may be beneficial to also investigate methods to regulate ΔFosB activity or to manage its downstream effects (21).

ΔFosB did not correspond to FSIQ in patients whose FSIQ is >85, suggesting that it may not closely reflect cognitive function in patients whose cognition scores are considered average or better. It is possible that ΔFosB expression is not sensitive enough to reflect more subtle variations in cognition. Indeed, in our previous study with postmortem tissue, ΔFosB did not correspond to MMSE scores in control individuals, who had average cognition, but did correspond to MMSE scores in MCI individuals, who have below average cognition (25). Similarly, ΔFosB expression corresponded to performance in a hippocampal-dependent memory task in mice used to study AD neuropathology, but not in wildtype control mice (19). Another possibility is that availability of binding partners for ΔFosB may be differentially expressed in the patient subgroups. ΔFosB, like other members of the AP-1 transcription factor family, usually form heterodimers with other AP-1 transcription factors, and the resulting complex regulates gene transcription (18, 56). Future research investigating whether binding partners of ΔFosB are expressed differently in patients with FSIQ above or below 85 may shed light on this possibility.

We also noted that ΔFosB did not correspond to individual WISC index scores, although this may in part be due to reduced power given variable sample sizes, since index scores were not available for all patients in the cohort. Interestingly, while most indices showed no trend or a negative trend with increasing magnitude of ΔFosB expression, the processing speed index instead showed a positive trend in multiple patient subcategories (Supplementary Figures 3–5). Higher processing speed has been hypothesized to reduce the demand on working memory capabilities (57, 58). Therefore, one possibility is that higher processing speed may be a compensatory mechanism engaged as a response to impaired working memory, which may be of interest for future investigations.

There were limitations in this study related to incomplete patient profiles, which may have precluded further insights. Seizure frequency is a critical piece of information that was unavailable for 14 of the 33 total patients investigated in this study. Even for the 19 patients for which this information was available, it is unclear when seizure frequency was assessed relative to when surgical resection of the hippocampus took place. The 8-day *in vivo* half-life of ΔFosB likely limits its ability to reflect seizure history beyond a few weeks prior to sample collection. Thus, ΔFosB may not closely track seizure frequency if that information was obtained too far in advance of the resection. Because it is not possible to obtain similarly processed hippocampal resection tissues from control individuals without a history of seizures, it was also not possible for us to determine the extent to which ΔFosB expression was increased above baseline at the time of surgery. In addition, the interval of time between WISC assessment and surgical resection of the hippocampus varied between patients, which could limit how closely ΔFosB expression (indicative of brain state at time of surgery) reflects cognitive performance (indicative of brain state at time of neuropsychiatric testing). It is also unclear what specific medications or other treatments patients had received prior to neuropsychiatric testing or hippocampectomy. Certain anti-seizure medications have been documented to have side effects on cognition and mood, which could affect performance during neuropsychiatric testing independently of ΔFosB (7, 59–62). Anti-seizure medication may also affect ΔFosB expression by altering seizure frequency (19, 63) or perhaps by direct regulation (21).

There is also limited information available about the etiology of seizures or which brain areas other than the hippocampus were affected by seizure activity, which are factors that can affect the extent and severity of cognitive impairment in epilepsy (7). In the present study, we investigated DG ΔFosB expression, which is indicative of seizure activity in the hippocampus itself, since ΔFosB accumulation occurs in neurons that are (hyper) active. However, ΔFosB in the DG does not regulate all domains of cognitive function, and seizures and lesions present in extra-hippocampal regions of the brain may also contribute to the variability in neuropsychiatric test performance. Indeed, some patients also had neurological comorbidities that could also impair cognition independently of or concurrently with seizures in the hippocampus. The presence of a tumor, for example, can directly disrupt local neural processing, and treatments for patients with tumors also often have negative effects on cognition (64). Cortical infarct resulting from ischemia can also induce neuronal excitotoxicity and cell death, loss of dendritic spines, alterations in synaptic receptor composition, and long-term potentiation deficits, which can all contribute to cognitive impairment (65). Indeed, there may be pathophysiological mechanisms that both increase seizure propensity and impair cognitive function (7, 66). These factors could obfuscate the relationship between DG ΔFosB expression and cognitive performance.

Despite these limitations, our study demonstrates that robust ΔFosB expression in the DG can be found in individuals of a broad range of ages and with varying medical conditions. Moreover, in specific subsets of those patient populations, DG ΔFosB expression corresponds to aspects of cognitive function, similar to rodent models of the same diseases. These findings suggest that ΔFosB pathways may be important for future studies to further elucidate, as understanding its mechanisms of action has the potential to create new avenues for therapeutic development.

## Data availability statement

The original contributions presented in the study are included in the article/Supplementary material, further inquiries can be directed to the corresponding authors.

## Ethics statement

The studies involving humans were approved by Baylor College of Medicine IRB protocol H-10255 and Children's Hospital of Philadelphia IRB protocol 19-016521. The studies were conducted in accordance with the local legislation and institutional requirements. The human samples used in this study were acquired from a by-product of routine care or industry. Written informed consent for participation was not required from the participants or the participants' legal guardians/next of kin in accordance with the national legislation and institutional requirements.

## Author contributions

C-HF: Conceptualization, Formal analysis, Investigation, Visualization, Writing – original draft, Writing – review & editing. JY: Formal analysis, Funding acquisition, Investigation, Writing – review & editing. CM: Investigation, Writing – review & editing. RR: Investigation, Writing – review & editing. DY: Investigation, Writing – review & editing. AV: Conceptualization, Formal analysis, Investigation, Project administration, Supervision, Writing – original draft, Writing – review & editing. JC: Conceptualization, Funding acquisition, Project administration, Supervision, Writing – original draft, Writing – review & editing.
