# Supplementary material for: Hippocampal ΔFosB expression is associated with cognitive impairment in a subgroup of patients with childhood epilepsies
Source: Front Neurol. 2024 Jan 11;14:1331194. doi: 10.3389/fneur.2023.1331194 (PMC10808715; doi:10.3389/fneur.2023.1331194)
Supplement: Supplementary file 1 [file Data_Sheet_1.pdf]

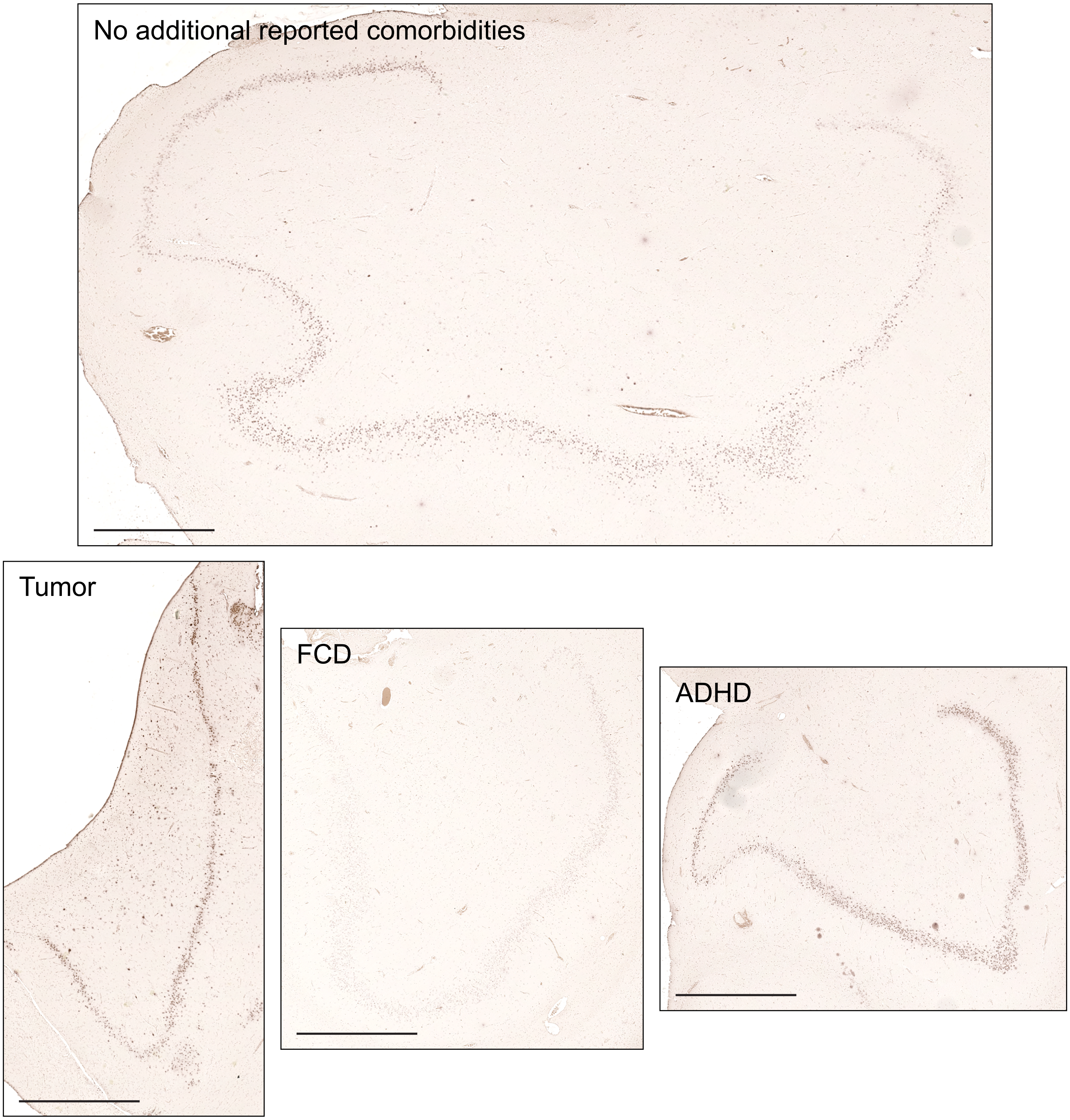

Supplementary Figure S1. Low magnification images of dentate gyrus ΔFosB immunoreactivity in patients with childhood epilepsies. Example images of dentate gyrus ΔFosB immunoreactivity in surgically resected hippocampal tissue from four different patients with childhood epilepsies who presented with or without additional reported comorbidities. FCD, focal cortical dysplasia; ADHD, attention-deficit/hyperactivity disorder. Scale bar: 1mm.

Condition		N	Average ± SD	P-value
All		33	45.44 ± 24.60	N/A
Sex	Male	16	39.27 ± 22.25	0.165
	Female	17	51.25 ± 25.92	
Age at surgery (years)	< 6	2	57.42 ± 49.01	0.568
	6 - 12	12	43.89 ± 23.70	
	12 - 18	16	48.25 ± 24.49	
	> 18	3	28.71 ± 15.80	
Encephalitis	Yes	5	45.50 ± 19.91	0.995
	No	28	45.43 ± 25.66	
Tumor	Yes	5	51.28 ± 30.96	0.573
	No	28	44.40 ± 23.82	
Infarction	Yes	4	34.06 ± 18.20	0.331
	No	29	47.01 ± 25.20	
FCD	Yes	3	23.92 ± 15.34	0.113
	No	20	47.59 ± 24.47	
Sturge-Weber syndrome	Yes	1	28.24 ± 0	N/A
	No	32	45.98 ± 24.79	
ADHD	Yes	8	50.67 ± 25.49	0.499
	No	25	43.77 ± 24.60	
Asperger's syndrome	Yes	1	40.72 ± 0	N/A
	No	32	45.59 ± 24.97	
Any complications	Yes	23	44.15 ± 23.41	0.655
	No	10	48.41 ± 28.24	

Supplementary Figure S2. Average dentate gyrus ΔFosB expression in different patient sub-groups. ΔFosB immunoreactivity was quantified in arbitrary units and averaged for patients grouped into different conditions. For each condition, ΔFosB expression was compared between patients from different subgroups of that condition, and p-value is indicated when applicable. P-value was calculated via two-tailed unpaired Student’s t-test for comparisons between two subgroups, and via one-way ANOVA for comparison between four subgroups (age). SD, standard deviation; FCD, focal cortical dysplasia; ADHD, attention-deficit/hyperactivity disorder .

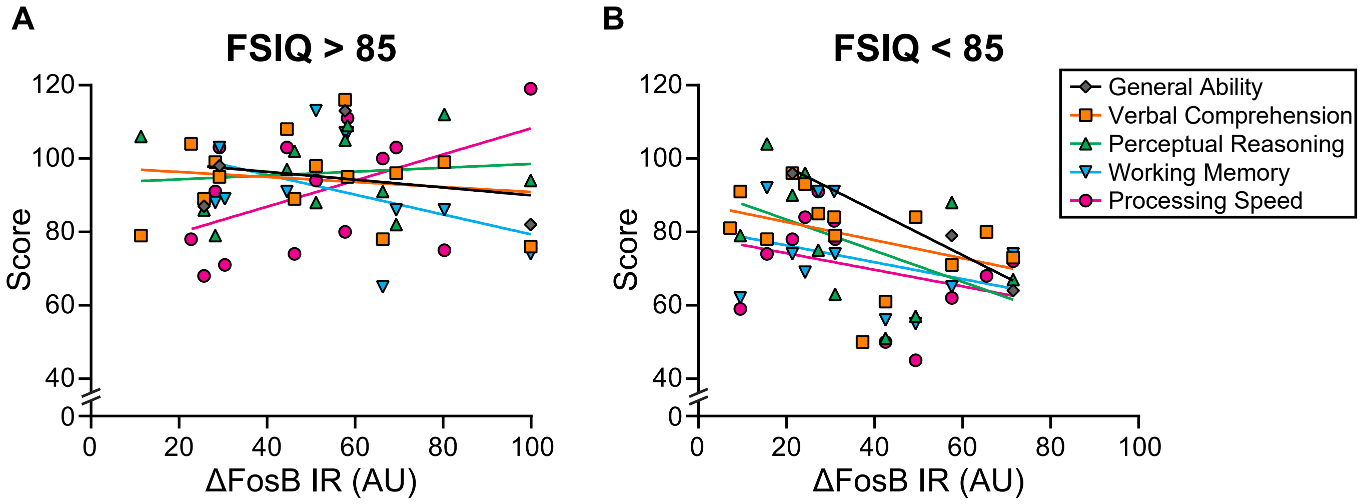

Supplementary Figure S3. Regression analyses of ΔFosB expression with WISC-IV index scores. (A-B) Regression analyses of dentate gyrus ΔFosB immunoreactivity (IR) with patient scores for general ability, verbal comprehension, perceptual reasoning, working memory, and processing speed indices in patients with FSIQ > 85 (A) and in patients with FSIQ < 85 (B). Note that not all index scores were available for every patient. FSIQ, Full-Scale Intelligence Quotient; AU, arbitrary units.

Condition		N	WISC (R^2; p-value)					
			FSIQ	GA	VC	PR	WM	PS
All Patients		33	0.037; 0.282	0.170; 0.358	0.001; 0.866	0.008; 0.685	0.000; 0.983	0.100; 0.115
Sex	Male	16	0.034; 0.497	0.570; 0.455	0.074; 0.348	0.001; 0.923	0.000; 0.964	0.006; 0.828
	Female	17	0.027; 0.532	0.191; 0.564	0.108; 0.252	0.050; 0.486	0.001; 0.916	0.219; 0.079
Time from NP test to surgery	≥ 3 mo	25	0.018; 0.5234	0.207; 0.546	0.002; 0.834	0.029; 0.488	0.011; 0.669	0.018; 0.570
	< 3 mo	8	0.032; 0.672	0.507; 0.496	0.038; 0.676	0.091; 0.622	0.407; 0.362	0.640; 0.056
Encephalitis	Yes	5	0.472; 0.200	N/A; N/A	0.653; 0.192	0.468; 0.520	0.070; 0.735	0.054; 0.706
	No	28	0.021; 0.459	0.086; 0.572	0.001; 0.892	0.016; 0.579	0.000; 0.972	0.111; 0.141
Tumor	Yes	5	0.541; 0.157	1; N/A	0.193; 0.561	0.371; 0.583	1; N/A	0.503; 0.291
	No	28	0.010; 0.608	0.231; 0.412	0.000; 0.954	0.023; 0.512	0.004; 0.796	0.135; 0.092
Infarction	Yes	4	0.000; 0.980	N/A; N/A	0.107; 0.674	0.000; 0.992	0.053; 0.771	0.011; 0.893
	No	29	0.066; 0.180	0.170; 0.358	0.004; 0.896	0.001; 0.897	0.019; 0.569	0.089; 0.178
FCD	Yes	3	0.598; 0.437	N/A; N/A	0.468; 0.520	N/A; N/A	N/A; N/A	N/A; N/A
	No	30	0.057; 0.204	0.170; 0.358	0.013; 0.594	0.004; 0.770	0.000; 0.947	0.116; 0.096
ADHD	Yes	8	0.080; 0.497	N/A; N/A	0.081; 0.642	1; N/A	1; N/A	0.030; 0.889
	No	25	0.016; 0.552	0.170; 0.358	0.014; 0.585	0.009; 0.672	0.000; 0.999	0.103; 0.135
No other complications		10	0.009; 0.800	0.210; 0.542	0.001; 0.924	0.010; 0.787	0.000; 0.987	0.158; 0.290
Hippocampal sclerosis	Yes	24	0.039; 0.353	0.219; 0.349	0.016; 0.594	0.003; 0.844	0.003; 0.848	0.254; 0.033
	No	9	0.035; 0.628	N/A; N/A	0.042; 0.628	0.014; 0.821	0.069; 0.615	0.036; 0.654

Supplementary Figure S4. Regression analysis statistics for comparing ΔFosB expression with WISC-IV scores in different patient subgroups. Dentate gyrus ΔFosB immunoreactivity was compared with WISC-IV scores with simple linear regression in different subgroups of patients. R^2 and p-values are indicated for each analysis. Note that not all index scores were available for every patient. N, number of patients within that specific subgroup; NP, neuropsychiatric; FCD, focal cortical dysplasia; ADHD, attention-deficit/hyperactivity disorder; FSIQ, Full-Scale Intelligence Quotient; GA, general ability; VC, verbal comprehension; PR, perceptual reasoning; WM, working memory; PS, processing speed.

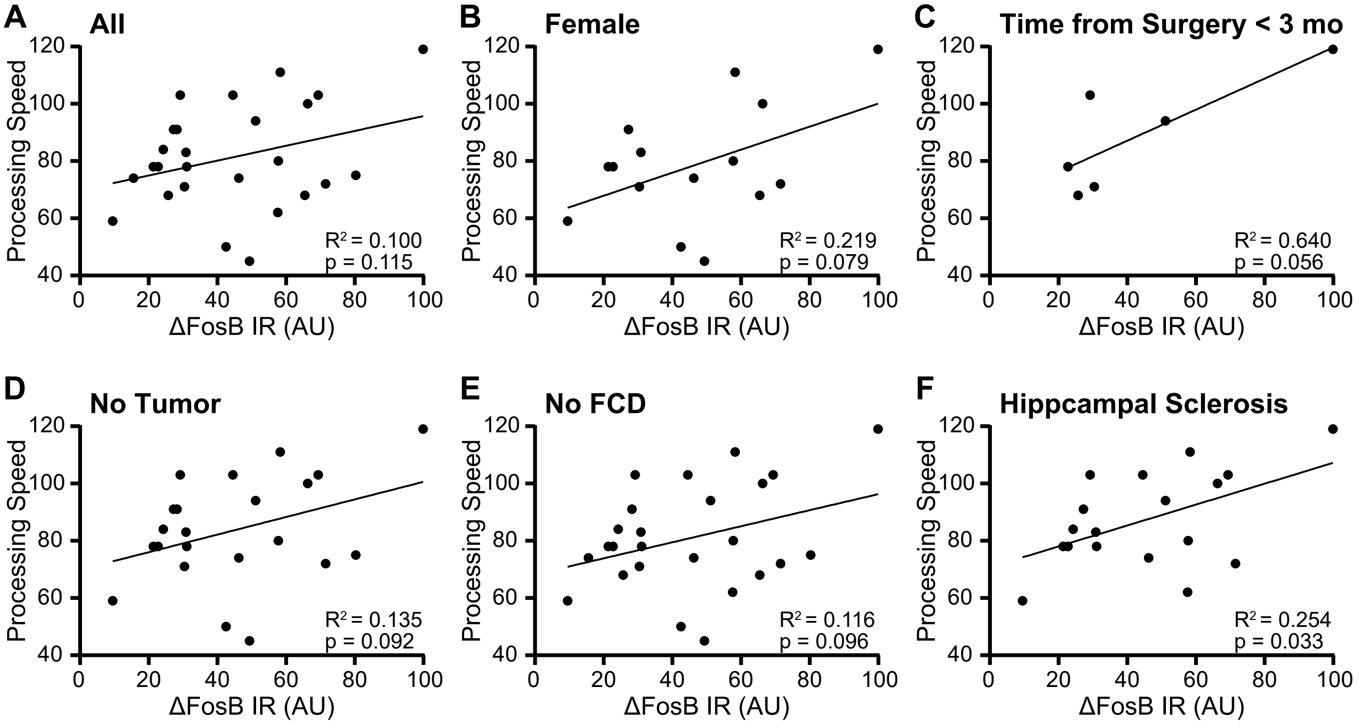

Supplementary Figure S5. Regression analyses of ΔFosB expression and processing speed index scores for patients with childhood epilepsies in different subgroups. (A-F) Regression analyses comparing dentate gyrus ΔFosB immunoreactivity (IR) with patient scores for the processing speed index in all patients (A), only female patients (B), only patients who received WISC-IV testing within 3 months of surgery (C), only patients without brain tumors (D), only patients without focal cortical dysplasia (FCD) (E), and only patients whose tissue showed hippocampal sclerosis (F). Note that not all index scores were available for every patient. AU, arbitrary unit.
